# Note on generalized Mittag-Leffler function

**DOI:** 10.1186/s40064-016-2299-x

**Published:** 2016-05-21

**Authors:** Rachana Desai, I. A. Salehbhai, A. K. Shukla

**Affiliations:** Department of Mathematics, K. J. Somaiya College of Engineering, Vidyavihar, Mumbai, 400077 India; Department of Mathematics, Government Engineering College, Bharuch, 392002 India; Department of Applied Mathematics & Humanities, S.V. National Institute of Technology, Surat, 395007 India

**Keywords:** Generalized Mittag-Leffler function, Fractional Calculus, 26A33, 33C20, 33E12

## Abstract

The present paper deals with the study of a generalized Mittag-Leffler function and associated fractional operator. The operator has been discussed in the space of Lebesgue measurable functions. The composition with Riemann–Liouville fractional integration operator has been obtained.

## Background

The well-known Mittag-Leffler function $E_{\alpha } (z)$ named after its originator, the Swedish mathematician Gosta Mittag-Leffler (1846–1927), is defined by (Mittag-Leffler 1903)

1 $$E_{\alpha } (z) = \sum\limits_{n = 0}^{\infty } {\frac{{z^{n} }}{\Gamma (\alpha n + 1)}} ; \, \quad z{\text{ is a complex variable and}}\,\text{Re} \left( \alpha \right) \ge 0.$$

The Mittag–Leffler function naturally occurs as the solution of fractional order differential equations. The various generalization of Mittag–Leffler function have been defined and studied by different authors.

Shukla and Prajapati (2007) introduced its generalization $E_{\alpha ,\beta }^{\gamma ,q} (z)$, this is defined as

2 $$E_{\alpha ,\beta }^{\gamma ,q} (z) = \sum\limits_{n = 0}^{\infty } {\frac{{(\gamma )_{qn} }}{\Gamma (\alpha n + \beta )}} \frac{{z^{n} }}{n!};$$

for $\alpha ,\beta ,\gamma \in C$; $\text{Re} \left( \alpha \right) > 0,\,\text{Re} \left( \beta \right) > 0,\text{Re} \left( \gamma \right) > 0,\text{Re} \left( \delta \right) > 0$, $q \in (0,1) \cup N$, and $(\gamma )_{qn} = \frac{\Gamma (\gamma + qn)}{\Gamma (\gamma )}$ denotes the generalized Pochhammer symbol.

Further, the generalization of (2) is also given by Khan and Ahmed (2013), as follows:

3 $$E_{\alpha ,\beta ,\upsilon ,\sigma ,\delta ,p}^{\mu ,\rho ,\gamma ,q} (z) = \sum\limits_{n = 0}^{\infty } {\frac{{(\mu )_{\rho n} (\gamma )_{qn} z^{n} }}{{\Gamma (\alpha n + \beta )(\upsilon )_{\sigma n} (\delta )_{pn} }}} ,$$

where $$\alpha ,\beta ,\gamma ,\delta ,\mu ,\upsilon \in C$$; $$p,q,\rho ,\sigma > 0$$;$$q \le Re(\alpha ) + p$$; $$\rho \le \text{Re} \left( \sigma \right) + p$$; $$q \le \text{Re} \left( \sigma \right) + p$$; $$\rho ,q \in (0,1) \cup N$$ and $$\hbox{min} \left( {\text{Re} \left( \alpha \right),\text{Re} \left( \beta \right), \, \text{Re} \left( \gamma \right),\text{Re} \left( \delta \right),\text{Re} \left( \mu \right),\text{Re} \left( \upsilon \right)} \right) > 0.$$

Here, the convergence conditions of (3) have been modified, which was given by Khan and Ahmed (2013).

The following well-known notations and definitions have been used:

Let $L(a,b)$ (Kilbas et al. 2004) be a set of all Lebesgue measurable real or complex valued functions $f(x)$ on $[a,b]$ i.e.

4 $$L(a,b) = \left\{ {f:\left\| f \right\|_{1} \equiv \int\limits_{a}^{b} {\left| {f(t)} \right|dt < \infty } } \right\}$$

Let $$f(x) \in L(a,b)$$, $$\mu \in C$$$$(\text{Re} (\mu ) > 0)$$ then the Riemann–Liouville left-sided fractional integrals of order $$\mu$$ (Miller and Ross 1993) is defined as

5 $${}_{a}I_{x}^{\mu } f(x) = I_{a + }^{\mu } f(x) = \frac{1}{\Gamma (\mu )}\int\limits_{a}^{x} {\frac{f(t)}{{(x - t)^{1 - \mu } }}} dt\,\,\,\,(x > a)$$

and the R–L right-sided fractional integral of order $\mu$ is defined as

6 $$_{b} I_{x}^{\mu } f\left( x \right) = I_{b - }^{\mu } f\left( x \right) = \frac{1}{\Gamma (\mu )\,}\int\limits_{x}^{b} {\frac{f(t)}{{(t - x)^{1 - \mu } }}} dt\,\,\,(x < b)$$

Miller and Ross (1993) defined the following:

If $$\mu ,\alpha ,\beta \in C$$, $$\text{Re} (\mu ) > 0$$; $$n = [\text{Re} (\mu )] + 1$$; $${\text{Re}} (\beta ) > 0$$ then

7 $$I_{a + }^{\mu } [(t - a)^{\beta - 1} ](x) = \frac{\Gamma (\beta )}{\Gamma (\mu + \beta )}(x - a)^{\mu + \beta - 1}$$

and

8 $$(D_{a + }^{\alpha } f)(x) = \left( {\frac{d}{dx}} \right)^{n} (I_{a + }^{n - \alpha } f)(x).$$

Khan and Ahmed (2013) proved the following result.

If $$\alpha ,\beta ,\gamma ,\delta ,\mu ,\upsilon ,\rho ,\sigma \in C$$; $$p,q > 0$$; $$q \le Re(\alpha ) + p$$ and

$$\text{Re} \left( \alpha \right) > 0,\text{Re} \left( \beta \right) > 0,\text{Re} \left( \gamma \right) > 0,\text{Re} \left( \delta \right) > 0,\text{Re} \left( \mu \right) > 0,\text{Re} \left( \upsilon \right) > 0,\text{Re} \left( \rho \right) > 0,\text{Re} \left( \sigma \right) > 0$$

then for $$m \in N,$$

9 $$\left( {\frac{d}{dz}} \right)^{m} \left[ {z^{\beta - 1} E_{\alpha ,\beta ,\upsilon ,\sigma ,\delta ,p}^{\mu ,\rho ,\gamma ,q} (wz^{\alpha } )} \right] = z^{\beta - m - 1} E_{\alpha ,\beta ,\upsilon ,\sigma ,\delta ,p}^{\mu ,\rho ,\gamma ,q} (wz^{\alpha } );\,\,\,\,\text{Re} \left( {\beta - m} \right) > 0.$$

In continuation of study, in this paper we give the operator associated with $E_{\alpha ,\beta ,\upsilon ,\sigma ,\delta ,p}^{\mu ,\rho ,\gamma ,q} (z)$ as follows:

Let $$f(x) \in L(a,b)$$, define

10 $$\left( {E_{\alpha ,\beta ,\upsilon ,\sigma ,\delta ,p;w;a + }^{\mu ,\rho ,\gamma ,q} f} \right)(x) = \int\limits_{a}^{x} {\left( {x - t} \right)^{\beta - 1} E_{\alpha ,\beta ,\upsilon ,\sigma ,\delta ,p}^{\mu ,\rho ,\gamma ,q} \left[ {w\left( {x - t} \right)^{\alpha } } \right]\,f\left( t \right)dt} ;\,\,\,\,x > a,$$

where $$\alpha ,\beta ,\gamma ,\delta ,\mu ,\upsilon \in C$$; $$p,q,\rho ,\sigma > 0$$; $$q \le Re(\alpha ) + p$$; $$\rho \le \text{Re} \left( \sigma \right) + p$$; $$q \le \text{Re} \left( \sigma \right) + p$$ and $$\hbox{min} \left( {\text{Re} \left( \alpha \right),\text{Re} \left( \beta \right),\text{Re} \left( \gamma \right),\text{Re} \left( \delta \right),\text{Re} \left( \mu \right),\text{Re} \left( \upsilon \right)} \right) > 0.$$

## Main results

Using the definition (4), one can easily prove following lemma.

### **Lemma 1**

*If*$\alpha ,\beta ,\gamma ,\delta ,\mu ,\upsilon ,\rho ,\sigma \in C$; $p,q > 0$; $q \le Re(\alpha ) + p$; $x > a$; $a \in R_{ + } = [0,\infty )$*and *$\hbox{min} \left( {\text{Re} \left( \alpha \right),\text{Re} \left( \beta \right),\text{Re} \left( \gamma \right),\text{Re} \left( \delta \right),\text{Re} \left( \mu \right),\text{Re} \left( \upsilon \right),\text{Re} \left( \rho \right),\text{Re} \left( \sigma \right)} \right) > 0,$*then*

11 $$E_{\alpha ,\beta ,\upsilon ,\sigma ,\delta ,p}^{\mu ,\rho ,\gamma ,q} (at^{\alpha } ) = \beta E_{\alpha ,\beta + 1,\upsilon ,\sigma ,\delta ,p}^{\mu ,\rho ,\gamma ,q} \left( {at^{\alpha } } \right) + \alpha tE_{\alpha ,\beta + 1,\upsilon ,\sigma ,\delta ,p}^{\mu ,\rho ,\gamma ,q} \left( {at^{\alpha } } \right)$$

### **Theorem 1**

*Let*$a \in R_{ + } = [0,\infty )$. *Let*$\alpha ,\beta ,\gamma ,\delta ,\mu ,\upsilon ,\rho ,\sigma \in C$; $p,q > 0$ ; $q \le Re(\alpha ) + p$*and*$\hbox{min} \left( {\text{Re} \left( \alpha \right),\text{Re} \left( \beta \right),\text{Re} \left( \gamma \right),\text{Re} \left( \delta \right),\text{Re} \left( \mu \right),\text{Re} \left( \upsilon \right),\text{Re} \left( \rho \right),\text{Re} \left( \sigma \right)} \right) > 0,\,\,\,x > a.$*Then*

12 $$\left( I_{a + }^{r} \left[(t - a)^{\beta - 1} E_{\alpha ,\beta ,\upsilon ,\sigma ,\delta ,p}^{\mu ,\rho ,\gamma ,q} \{ w(t - a)^{\alpha } \} \right)(x) = (x - a)^{r + \beta - 1} E_{\alpha ,\beta + r,\upsilon ,\sigma ,\delta ,p}^{\mu ,\rho ,\gamma ,q} [w(x - a)^{\alpha } \right]$$

13 $$\left( D_{a + }^{r} \left[(t - a)^{\beta - 1} E_{\alpha ,\beta ,\upsilon ,\sigma ,\delta ,p}^{\mu ,\rho ,\gamma ,q} \{ w(t - a)^{\alpha } \} \right)(x) = (x - a)^{\beta - r - 1} E_{\alpha ,\beta - r,\upsilon ,\sigma ,\delta ,p}^{\mu ,\rho ,\gamma ,q} [w(x - a)^{\alpha } \right]$$

### Proof

Using definitions (3) and (5) and further simplification gives

$$\begin{aligned} & \left( I_{a + }^{r} \left[(t - a)^{\beta - 1} E_{\alpha ,\beta ,\upsilon ,\sigma ,\delta ,p}^{\mu ,\rho ,\gamma ,q} \{ w(t - a)^{\alpha } \} \right)(x)\right. \\ & {\kern 1pt} \quad = \sum\limits_{n = 0}^{\infty } {\frac{{(\mu )_{\rho n} (\gamma )_{qn} }}{\Gamma (\alpha n + \beta )}\frac{{w^{n} }}{{(\upsilon )_{\sigma n} (\delta )_{pn} }}} \left( {I_{a + }^{r} [(t - a)^{\alpha n + \beta - 1} ]} \right)(x) \\ & \quad = \sum\limits_{n = 0}^{\infty } {\frac{{(\mu )_{\rho n} (\gamma )_{qn} }}{\Gamma (\alpha n + \beta )}\frac{{w^{n} }}{{(\upsilon )_{\sigma n} (\delta )_{pn} }}} \frac{\Gamma (\alpha n + \beta )}{\Gamma (\alpha n + \beta + r)}(x - a)^{\alpha n + \beta + r - 1} \\ & \quad = (x - a)^{r + \beta - 1} \sum\limits_{n = 0}^{\infty } {\frac{{(\mu )_{\rho n} (\gamma )_{qn} }}{\Gamma (\alpha n + \beta + r)}} \frac{{[w(x - a)^{\alpha } ]}}{{(\upsilon )_{\sigma n} (\delta )_{pn} }}^{n} \\ & \quad =\left. (x - a)^{r + \beta - 1} E_{\alpha ,\beta + r,\upsilon ,\sigma ,\delta ,p}^{\mu ,\rho ,\gamma ,q} [w(x - a)^{\alpha } \right]. \\ \end{aligned}$$

This completes the proof of (12).□

To prove (13), we use definitions (8) and further simplification gives

$$\begin{aligned} & \left( D_{a + }^{r} \left[(t - a)^{\beta - 1} E_{\alpha ,\beta ,\upsilon ,\sigma ,\delta ,p}^{\mu ,\rho ,\gamma ,q} \{ w(t - a)^{\alpha } \} \right)(x)\right. \\ & \quad = \left( {\frac{d}{dx}} \right)^{n} \left( {I_{a + }^{n - r} [(t - a)^{\beta - 1} E_{\alpha ,\beta ,\upsilon ,\sigma ,\delta ,p}^{\mu ,\rho ,\gamma ,q} \{ w(t - a)^{\alpha } \} ]} \right)(x), \\ \end{aligned}$$

On applying (12) with replacement of $r$ by $n - r$, the above equation reduces to

$$= \left( {\frac{d}{dx}} \right)^{n} \left[(x - a)^{\beta + n - r - 1} E_{\alpha ,\beta + n - r,\upsilon ,\sigma ,\delta ,p}^{\mu ,\rho ,\gamma ,q} \{ w(x - a)^{\alpha } \} \right],$$

From (9), we get

$$\begin{aligned} &= (x - a)^{\beta + n - r - 1 - n} E_{\alpha ,\beta + n - r - n,\upsilon ,\sigma ,\delta ,p}^{\mu ,\rho ,\gamma ,q} \{ w(x - a)^{\alpha } \} \hfill \\ &= (x - a)^{\beta - r - 1} E_{\alpha ,\beta - r,\upsilon ,\sigma ,\delta ,p}^{\mu ,\rho ,\gamma ,q} [w(x - a)^{\alpha } ]. \hfill \\ \end{aligned}$$

### **Theorem 2**

*Let*$$a \in R_{ + } = [0,\infty ),$$$$\alpha ,\beta ,\gamma ,\delta ,\mu ,\upsilon ,\rho ,\sigma \in C$$; $$p,q > 0$$*and*$$q \le Re(\alpha ) + p$$*and*$$\hbox{min} \left( {\text{Re} \left( \alpha \right),\text{Re} \left( \beta \right),\text{Re} \left( \gamma \right),\text{Re} \left( \delta \right),\text{Re} \left( \mu \right),\text{Re} \left( \upsilon \right),\text{Re} \left( \rho \right),\text{Re} \left( \sigma \right)} \right) > 0$$$$x > a.$$*Then*

14 $$\left( {E_{\alpha ,\beta ,\upsilon ,\sigma ,\delta ,p;w;a + }^{\mu ,\rho ,\gamma ,q} (t - a)^{r - 1} } \right)(x) = (x - a)^{\beta + r - 1} \Gamma (r)E_{\alpha ,\beta + r,\upsilon ,\sigma ,\delta ,p}^{\mu ,\rho ,\gamma ,q} \left( {w(x - a)^{\alpha } } \right).$$

### Proof

Taking $f(t) = (t - a)^{r - 1}$ in (10), we get

$$\begin{aligned} \left( {E_{\alpha ,\beta ,\upsilon ,\sigma ,\delta ,p;w;a + }^{\mu ,\rho ,\gamma ,q} (t - a)^{r - 1} } \right)(x) & = \int\limits_{a}^{x} {(x - t)^{\beta - 1} E_{\alpha ,\beta ,\upsilon ,\sigma ,\delta ,p}^{\mu ,\rho ,\gamma ,q} [w(x - t)^{\alpha } ](t - a)^{r - 1} dt} \\ & = \sum\limits_{n = 0}^{\infty } {\frac{{(\mu )_{\rho n} (\gamma )_{qn} }}{\Gamma (\alpha n + \beta )}\frac{{w^{n} }}{{(\upsilon )_{\sigma n} (\delta )_{pn} }}} \int\limits_{a}^{x} {(x - t)^{\alpha n + \beta - 1} (t - a)^{r - 1} dt} , \\ \end{aligned}$$

Replacing $t$ by $a + (x - a)t$ and simplifying the above equation

$$= \sum\limits_{n = 0}^{\infty } {\frac{{(\mu )_{\rho n} (\gamma )_{qn} }}{\Gamma (\alpha n + \beta )}\frac{{w^{n} }}{{(\upsilon )_{\sigma n} (\delta )_{pn} }}} (x - a)^{\alpha n + \beta + r - 1} B(\alpha n + \beta ,r)$$

and further simplification of above equation gives the proof of Theorem 2.

### **Theorem 3**

*Let*$$a \in R_{ + } = [0,\infty ),$$$$\alpha ,\beta ,\gamma ,\delta ,\mu ,\upsilon ,\rho ,\sigma \in C$$; $$p,q > 0$$*and*$$q \le Re(\alpha ) + p$$*and*$$\hbox{min} \left( {\text{Re} \left( \alpha \right),\text{Re} \left( \beta \right),\text{Re} \left( \gamma \right),\text{Re} \left( \delta \right),\text{Re} \left( \mu \right),\text{Re} \left( \upsilon \right),\text{Re} \left( \rho \right),\text{Re} \left( \sigma \right)} \right) > 0,$$$$b > a.$$, *Then the operator*$$E_{\alpha ,\beta ,\upsilon ,\sigma ,\delta ,p;w;a + }^{\mu ,\rho ,\gamma ,q}$$*is bounded on*$$L(a,b)$$*and*

15 $$\left\| {E_{\alpha ,\beta ,\upsilon ,\sigma ,\delta ,p;w;a + }^{\mu ,\rho ,\gamma ,q} f} \right\|_{1} \le B\left\| f \right\|_{1} ,$$

*where*

16 $$B = (b - a)^{{\text{Re} (\beta )}} \sum\limits_{n = 0}^{\infty } {\frac{{\left| {(\mu )_{\rho n} } \right|\left| {(\gamma )_{qn} } \right|}}{{[\text{Re} (\alpha )n + \text{Re} (\beta )]\left| {\Gamma (\alpha n + \beta )} \right|}}} \frac{{\left| {w(b - a)^{{\text{Re} (\alpha )}} } \right|^{n} }}{{\left| {(\upsilon )_{\sigma n} } \right|\left| {(\delta )_{pn} } \right|}}.$$

### Proof

On using the definition (10) and applying Dirichlet’s formula (Samko et al. 1993), we have

$$\begin{aligned} \left\| {E_{\alpha ,\beta ,\upsilon ,\sigma ,\delta ,p;w;a + }^{\mu ,\rho ,\gamma ,q} f} \right\|_{1} & = \int\limits_{a}^{b} {\left| {\int\limits_{a}^{x} {(x - t)^{\beta - 1} } E_{\alpha ,\beta ,\upsilon ,\sigma ,\delta ,p}^{\mu ,\rho ,\gamma ,q} [w(x - t)^{\alpha } ]\,f(t)dt} \right|} dx \\ & \le \int\limits_{a}^{b} {\left[ {\int\limits_{t}^{b} {(x - t)^{{\text{Re} (\beta ) - 1}} } \left| {E_{\alpha ,\beta ,\upsilon ,\sigma ,\delta ,p}^{\mu ,\rho ,\gamma ,q} [w(x - t)^{\alpha } ]} \right|dx} \right]\left| {f(t)} \right|} dt, \\ \end{aligned}$$

Taking $u = x - t$ in inner integral, this yields

$$\begin{aligned} \left\| {E_{\alpha ,\beta ,\upsilon ,\sigma ,\delta ,p;w;a + }^{\mu ,\rho ,\gamma ,q} f} \right\|_{1} & \le \int\limits_{a}^{b} {\left[ {\int\limits_{0}^{b - t} {u^{{\text{Re} (\beta ) - 1}} } \left| {E_{\alpha ,\beta ,\upsilon ,\sigma ,\delta ,p}^{\mu ,\rho ,\gamma ,q} [wu^{\alpha } ]} \right|du} \right]\left| {f(t)} \right|} dt \\ & \le \int\limits_{a}^{b} {\sum\limits_{n = 0}^{\infty } {\frac{{\left| {(\mu )_{\rho n} } \right|\left| {(\gamma )_{qn} } \right|}}{{\left| {\Gamma (\alpha n + \beta )} \right|}}\frac{{\left| w \right|^{n} }}{{\left| {(\upsilon )_{\sigma n} } \right|\left| {(\delta )_{pn} } \right|}}\left[ {\frac{{u^{{\text{Re} (\alpha )n + \text{Re} (\beta )}} }}{{\text{Re} (\alpha )n + \text{Re} (\beta )}}} \right]_{0}^{b - a} } \left| {f(t)} \right|} dt \\ & = (b - a)^{{\text{Re} (\beta )}} \sum\limits_{n = 0}^{\infty } {\frac{{\left| {(\mu )_{\rho n} } \right|\left| {(\gamma )_{qn} } \right|}}{{\left| {\Gamma (\alpha n + \beta )} \right|}}\frac{{\left| w \right|^{n} }}{{\left| {(\upsilon )_{\sigma n} } \right|\left| {(\delta )_{pn} } \right|}}\frac{{(b - a)^{{\text{Re} (\alpha )n}} }}{{[\text{Re} (\alpha )n + \text{Re} (\beta )]}}} \int\limits_{a}^{b} {\left| {f(t)} \right|dt} \\ \end{aligned}$$

This completes the proof.□

### **Theorem 4**

(Composition with Riemann–Liouville fractional integration operator) *Let*$\alpha ,\beta ,\gamma ,\delta ,\mu ,\upsilon ,\rho ,\sigma \in C$; $p,q > 0$; $q \le Re(\alpha ) + p$; $b > a$*and*$\hbox{min} \left( {\text{Re} \left( \alpha \right),\text{Re} \left( \beta \right),\text{Re} \left( \gamma \right),\text{Re} \left( \delta \right),\text{Re} \left( \mu \right),\text{Re} \left( \upsilon \right),\text{Re} \left( \rho \right),\text{Re} \left( \sigma \right)} \right) > 0.$*Then the relation*

17 $$I_{a + }^{r} E_{\alpha ,\beta ,\upsilon ,\sigma ,\delta ,p;w;a + }^{\mu ,\rho ,\gamma ,q} f \equiv E_{\alpha ,\beta + r,\upsilon ,\sigma ,\delta ,p;w;a + }^{\mu ,\rho ,\gamma ,q} f \equiv E_{\alpha ,\beta ,\upsilon ,\sigma ,\delta ,p;w;a + }^{\mu ,\rho ,\gamma ,q} I_{a + }^{r} f$$

*holds for any summable function*$f \in L(a,b).$

### Proof

From (10) and (5), we get

$$(I_{a + }^{r} E_{\alpha ,\beta ,\upsilon ,\sigma ,\delta ,p;w;a + }^{\mu ,\rho ,\gamma ,q} f)(x) = \frac{1}{\Gamma (r)}\int\limits_{a}^{x} {\int\limits_{a}^{u} {(x - u)^{r - 1} (u - t)^{\beta - 1} } E_{\alpha ,\beta ,\upsilon ,\sigma ,\delta ,p}^{\mu ,\rho ,\gamma ,q} [w(u - t)^{\alpha } ]f(t)dt} du$$

Applying Dirichlet’s formula (Samko et al. 1993), we get

$$= \int\limits_{a}^{x} {\left[ {\frac{1}{\Gamma (r)}\int\limits_{t}^{x} {(x - u)^{r - 1} (u - t)^{\beta - 1} } E_{\alpha ,\beta ,\upsilon ,\sigma ,\delta ,p}^{\mu ,\rho ,\gamma ,q} [w(u - t)^{\alpha } ]du} \right]} f(t)dt$$

Substituting $u - t = \tau$ in the above equation, we get

$$= \int\limits_{a}^{x} {\left[ {\frac{1}{\Gamma (r)}\int\limits_{0}^{x - t} {(x - t - \tau )^{r - 1} \tau^{\beta - 1} } E_{\alpha ,\beta ,\upsilon ,\sigma ,\delta ,p}^{\mu ,\rho ,\gamma ,q} [w\tau^{\alpha } ]d\tau } \right]} f(t)dt$$

Again using (5), this equation becomes

$$= \int\limits_{a}^{x} {\left( {I_{0 + }^{\mu } \left[ {\tau^{\beta - 1} E_{\alpha ,\beta }^{\gamma ,q} (w\tau^{\alpha } )\,} \right]} \right)} \,(x - t)\,f(t)dt,$$

Applying (12), this yields

$$= \int\limits_{a}^{x} {(x - t)^{\beta + r - 1} E_{\alpha ,\beta + r,\upsilon ,\sigma ,\delta ,p}^{\mu ,\rho ,\gamma ,q} [w(x - t)^{\alpha } ]\,f(t)dt} ,$$

Using (10), we get

$$= (E_{\alpha ,\beta + r,\upsilon ,\sigma ,\delta ,p;w;a + }^{\mu ,\rho ,\gamma ,q} f)(x).$$

The other equality can also be proved in the similar way.

### **Theorem 5**

*Let*$\alpha ,\beta ,\gamma ,\delta ,\mu ,\upsilon ,\rho ,\sigma \in C$; $p,q > 0$; $q \le Re(\alpha ) + p$; $b > a$*and*$\hbox{min} \left( {\text{Re} \left( \alpha \right),\text{Re} \left( \beta \right),\text{Re} \left( \gamma \right),\text{Re} \left( \delta \right),\text{Re} \left( \mu \right),\text{Re} \left( \upsilon \right),\text{Re} \left( \rho \right),\text{Re} \left( \sigma \right)} \right) > 0.$*Then the relation*

18 $$D_{a + }^{r} E_{\alpha ,\beta ,\upsilon ,\sigma ,\delta ,p;w;a + }^{\mu ,\rho ,\gamma ,q} f \equiv E_{\alpha ,\beta - r,\upsilon ,\sigma ,\delta ,p;w;a + }^{\mu ,\rho ,\gamma ,q} f$$

*holds for any continuous function*$f \in C[a,b]$.

### Proof

From (8), we have

$$\left( D_{a + }^{r} E_{\alpha ,\beta ,\upsilon ,\sigma ,\delta ,p;w;a + }^{\mu ,\rho ,\gamma ,q} f \right)(x) = \left( {\frac{d}{dx}} \right)^{n} \left( {I_{a + }^{n - r} E_{\alpha ,\beta ,\upsilon ,\sigma ,\delta ,p;w;a + }^{\mu ,\rho ,\gamma ,q} f} \right)(x)$$

Again using Theorem 4 and definition (10),

19 $$= \left( {\frac{d}{dx}} \right)^{n} \left[ {\int\limits_{a}^{x} {(x - t)^{\beta + n - r - 1} } E_{\alpha ,\beta + n - r,\upsilon ,\sigma ,\delta ,p}^{\mu ,\rho ,\gamma ,q} [w(x - t)^{\alpha } ]f(t)dt} \right]$$

The integrand in the above equation is continuous function on $[a,b]$, here we take

20 $$\frac{d}{dx}\int\limits_{a}^{x} {h(x,t)dt = \int\limits_{a}^{x} {\frac{\partial }{\partial x}h(x,t)dt + h(x,x)} }$$

From (19) and (20), we get

$$\left( D_{a + }^{r} E_{\alpha ,\beta ,\upsilon ,\sigma ,\delta ,p;w;a + }^{\mu ,\rho ,\gamma ,q} f \right)(x) = \left( {\frac{d}{dx}} \right)^{n - 1} \int\limits_{a}^{x} {(x - t)^{\beta + n - r - 2} } E_{\alpha ,\beta + n - r - 1,\upsilon ,\sigma ,\delta ,p}^{\mu ,\rho ,\gamma ,q} [w(x - t)^{\alpha } ]f(t)dt$$

Applying same procedures as above, this led the proof of the theorem. This is easy to prove by using mathematical induction method also.

### **Theorem 6**

*Let*$$a \in R_{ + } = [0,\infty ),$$$$\alpha ,\beta ,\gamma ,\delta ,\mu ,\upsilon ,\rho ,\sigma \in C$$; $$p,q > 0;$$$$q \le Re(\alpha ) + p$$*and*$$\hbox{min} \left( {\text{Re} \left( \alpha \right),\text{Re} \left( \beta \right),\text{Re} \left( \gamma \right),\text{Re} \left( \delta \right),\text{Re} \left( \mu \right),\text{Re} \left( \upsilon \right),\text{Re} \left( \rho \right),\text{Re} \left( \sigma \right)} \right) > 0,\,\,\,\,x > a.$$*Then*

21 $$\left( I_{0 + }^{r} \left[t^{\beta - 1} E_{\alpha ,\beta ,\upsilon ,\sigma ,\delta ,p}^{\mu ,\rho ,\gamma ,q} \{ at^{\alpha } \} \right)(x) = x^{r + \beta - 1} E_{\alpha ,\beta + r,\upsilon ,\sigma ,\delta ,p}^{\mu ,\rho ,\gamma ,q} [ax^{\alpha } ] \right.$$

### Proof

We have

$$\begin{aligned} \left( I_{0 + }^{r} \left[t^{\beta - 1} E_{\alpha ,\beta ,\upsilon ,\sigma ,\delta ,p}^{\mu ,\rho ,\gamma ,q} \{ at^{\alpha } \} \right. \right)(x) & = \sum\limits_{n = 0}^{\infty } {\frac{{(\mu )_{\rho n} (\gamma )_{qn} }}{\Gamma (\alpha n + \beta )}\frac{{a^{n} }}{{(\upsilon )_{\sigma n} (\delta )_{pn} }}} \left( {I_{0 + }^{r} [t^{\alpha n + \beta - 1} ]} \right)(x) \\ & = \sum\limits_{n = 0}^{\infty } {\frac{{(\mu )_{\rho n} (\gamma )_{qn} }}{\Gamma (\alpha n + \beta )}\frac{{a^{n} }}{{(\upsilon )_{\sigma n} (\delta )_{pn} }}} \frac{\Gamma (\alpha n + \beta )}{\Gamma (\alpha n + \beta + r)}(x)^{\alpha n + \beta + r - 1} \\ & = x^{r + \beta - 1} \sum\limits_{n = 0}^{\infty } {\frac{{(\mu )_{\rho n} (\gamma )_{qn} }}{\Gamma (\alpha n + \beta + \mu )}} \frac{{[ax^{\alpha } ]^{n} }}{{(\upsilon )_{\sigma n} (\delta )_{pn} }} \\ & = x^{r + \beta - 1} E_{\alpha ,\beta + r,\upsilon ,\sigma ,\delta ,p}^{\mu ,\rho ,\gamma ,q} [ax^{\alpha } ]. \\ \end{aligned}$$

This completes the proof.□

### **Corollary 1**

*If*$$\alpha ,\beta ,\gamma ,\delta ,\mu ,\upsilon ,\rho ,\sigma \in C$$; $$p,q > 0$$*and*$$q \le Re(\alpha ) + p$$$$x > a$$; $$\hbox{min} \left( {\text{Re} \left( \alpha \right),\text{Re} \left( \beta \right),\text{Re} \left( \gamma \right),\text{Re} \left( \delta \right),\text{Re} \left( \mu \right),\text{Re} \left( \upsilon \right),\text{Re} \left( \rho \right),\text{Re} \left( \sigma \right)} \right) > 0,\,\,\,\,a \in R_{ + } = [0,\infty ).$$* Let*$$I_{0 + }^{r}$$*be the left-sided operator of Riemann–Liouville fractional integral. Then*

22 $$\left( I_{0 + }^{r} \left[ t^{\beta - 1} E_{\alpha ,\beta ,\upsilon ,\sigma ,\delta ,p}^{\mu ,\rho ,\gamma ,q} \{ at^{\alpha } \} \right)(x)\right. = x^{r + \beta - 1} \{ (\beta + r)E_{\alpha ,\beta + r + 1,\upsilon ,\sigma ,\delta ,p}^{\mu ,\rho ,\gamma ,q} + x\frac{d}{dx}E_{\alpha ,\beta + r + 1,\upsilon ,\sigma ,\delta ,p}^{\mu ,\rho ,\gamma ,q} (ax^{\alpha } )\}$$

Proof is very obvious from Lemma 1 and Theorem 6.

### **Theorem 7**

*Let*$$a \in R_{ + } = [0,\infty )$$, $$\alpha ,\beta ,\gamma ,\delta ,\mu ,\upsilon ,\rho ,\sigma \in C$$; $$p,q > 0;$$$$q \le Re(\alpha ) + p$$*and*$$\hbox{min} \left( {\text{Re} \left( \alpha \right),\text{Re} \left( \beta \right),\text{Re} \left( \gamma \right),\text{Re} \left( \delta \right),\text{Re} \left( \mu \right),\text{Re} \left( \upsilon \right),\text{Re} \left( \rho \right),\text{Re} \left( \sigma \right)} \right) > 0,\,\,\,x > a,$$$$I_{ - }^{r}$$*be the right-sided operator of Riemann–Liouville fractional integral. Then*

$$\left( {I_{ - }^{r} [t^{ - r - \beta } E_{\alpha ,\beta ,\upsilon ,\sigma ,\delta ,p}^{\mu ,\rho ,\gamma ,q} \{ at^{ - \alpha } \} } \right)(x) = x^{ - \beta } E_{\alpha ,\beta + r,\upsilon ,\sigma ,\delta ,p}^{\mu ,\rho ,\gamma ,q} [ax^{ - \alpha } ]$$

### Proof

Let

$$\left( {I_{ - }^{r} [t^{ - r - \beta } E_{\alpha ,\beta ,\upsilon ,\sigma ,\delta ,p}^{\mu ,\rho ,\gamma ,q} \{ at^{ - \alpha } \} } \right)(x) = \frac{1}{\Gamma (r)}\int\limits_{x}^{\infty } {(t - x)^{r - 1} t^{ - r - \beta } \sum\limits_{n = 0}^{\infty } {\frac{{(\mu )_{\rho n} (\gamma )_{qn} (at^{ - \alpha } )^{n} }}{{\Gamma (\alpha n + \beta )(\upsilon )_{\sigma n} (\delta )_{pn} }}} dt}$$

On changing the order of the summation and integration then afterward applying beta function, this gives

$$= x^{ - \beta } \sum\limits_{n = 0}^{\infty } {\frac{{(\mu )_{\rho n} (\gamma )_{qn} (ax^{ - \alpha } )^{n} }}{{\Gamma (\alpha n + \beta + r)(\upsilon )_{\sigma n} (\delta )_{pn} }}} = x^{ - \beta } E_{\alpha ,\beta + r,\upsilon ,\sigma ,\delta ,p}^{\mu ,\rho ,\gamma ,q} \{ ax^{ - \alpha } \}$$

### **Corollary 2**

*If*$$\alpha ,\beta ,\gamma ,\delta ,\mu ,\upsilon ,\rho ,\sigma \in C$$; $$p,q > 0$$*and*$$q \le Re(\alpha ) + p; \,\,\, x > a$$*;*$$\hbox{min} \left( {\text{Re} \left( \alpha \right),\text{Re} \left( \beta \right),\text{Re} \left( \gamma \right),\text{Re} \left( \delta \right),\text{Re} \left( \mu \right),\text{Re} \left( \upsilon \right),\text{Re} \left( \rho \right),\text{Re} \left( \sigma \right)} \right) > 0,$$$$a \in R_{ + } = [0,\infty )$$. *Let*$$I_{ - }^{r}$$*be the right-sided operator of Riemann–Liouville fractional integral. Then*

23 $$\left( {I_{ - }^{r} [t^{ - r - \beta } E_{\alpha ,\beta ,\upsilon ,\sigma ,\delta ,p}^{\mu ,\rho ,\gamma ,q} \{ at^{ - \alpha } \} } \right)(x) = x^{ - r - \beta } \{ (\beta + r)E_{\alpha ,\beta + r + 1,\upsilon ,\sigma ,\delta ,p}^{\mu ,\rho ,\gamma ,q} + x\frac{d}{dx}E_{\alpha ,\beta + r + 1,\upsilon ,\sigma ,\delta ,p}^{\mu ,\rho ,\gamma ,q} (ax^{ - \alpha } )\}$$

## Conclusion

In this paper, we proved some properties of generalized Mittag-Leffler functions and also used the fractional calculus approach to prove Theorems 4, 5, 6 and 7.
